# Unmasking Coronary Artery Disease With Intermittent Left Bundle Branch Block: A Case Report

**DOI:** 10.7759/cureus.54155

**Published:** 2024-02-13

**Authors:** Kawthar A Alabdrabalrasol, Amal S Al Sulaiman, Lama T Alkhunaizi, Sajedh N Al Kazim, Dunya Alfaraj

**Affiliations:** 1 Emergency, Imam Abdulrahman Bin Faisal University, King Fahad University Hospital, Dammam, SAU; 2 Internal Medicine, Imam Abdulrahman Bin Faisal University, King Fahad University Hospital, Dammam, SAU; 3 College of Medicine, Imam Abdulrahman Bin Faisal University, King Fahad University Hospital, Dammam, SAU; 4 General Practice, Imam Abdulrahman Bin Faisal University, King Fahad University Hospital, Dammam, SAU

**Keywords:** coronary artery disease (cad), chest pain, dyspnea, transient, intermittent left bundle branch block (lbbb)

## Abstract

Intermittent left bundle branch block (LBBB) is an unusual phenomenon, with very few cases documented in the literature. It is often considered a reflection of underlying conditions known to increase the risk of cardiovascular morbidity and death, including coronary artery disease (CAD), cardiomyopathy, hypertensive heart disease, and aortic valve disease. In rare instances, coronary vasospasm is the sole underlying condition. It is typically diagnosed by ECG and managed according to the underlying cause.

We describe a case of intermittent LBBB presenting with chest pain. The ECG showed dynamic changes with transient/intermittent LBBB. An angiogram was performed, revealing significant coronary lesions. The patient was eventually managed conservatively and discharged on dual antiplatelet therapy for a duration of one year with a one-month clinic follow-up where his condition improved.

Intermittent LBBB represents a transient disturbance in the intraventricular conduction system, where diseased conduction occurs secondary to an underlying cause, but normal conduction eventually restores. This results in complexes where LBBB appears alongside normally conducted beats in a single ECG tracing.

There is limited knowledge about the prognosis of patients with intermittent LBBB; therefore, patients with LBBB should undergo careful evaluation due to the known association with serious cardiac pathologies, particularly cardiac ischemia. It is important to consider the potential adverse effects on ventricular function.

## Introduction

Intermittent left bundle branch block (LBBB) is an unusual phenomenon. Although it was described decades ago, only a few cases have been reported in the literature. It encompasses various mechanisms and can arise from multiple underlying causes, yet the precise pathophysiological basis remains elusive.

LBBB often serves as an indicator of several underlying conditions associated with heightened cardiovascular morbidity and mortality. These encompass coronary artery disease (CAD), cardiomyopathy, hypertensive heart disease, and aortic valve disease. In rare instances, coronary vasospasm stands as the singular underlying condition. Intermittent LBBB manifests as a transient disruption in intraventricular conduction. It results in complexes where diseased conduction occurs, but ultimately, normal conduction is restored, presenting a combination of LBBB complexes alongside normally conducted beats in a single ECG tracing [1].

## Case presentation

We present the case of a 60-year-old Saudi female with a background of type II diabetes and hypertension. She presented to the emergency department with chest pain persisting for two days. The pain was described as severe retrosternal discomfort, not associated with physical activity, radiating to the left arm, and lasting for 20 to 30 minutes. This was accompanied by profuse diaphoresis, nausea, and dyspnea. The patient denied any history of smoking, alcohol consumption, or illicit drug use.

On physical examination, she appeared well, alert, conscious, and oriented. The patient was hemodynamically stable, with a blood pressure of 160/80 mmHg, a pulse rate of 70 bpm, and normal oxygen saturation on room air. The cardiovascular exam revealed normal heart sounds with a regular rhythm. There was no jugular venous distention. Lung auscultation revealed clear breath sounds. Peripheral pulses were strong and equal in all limbs, and the lower extremities showed no signs of edema.

**Figure 1:**
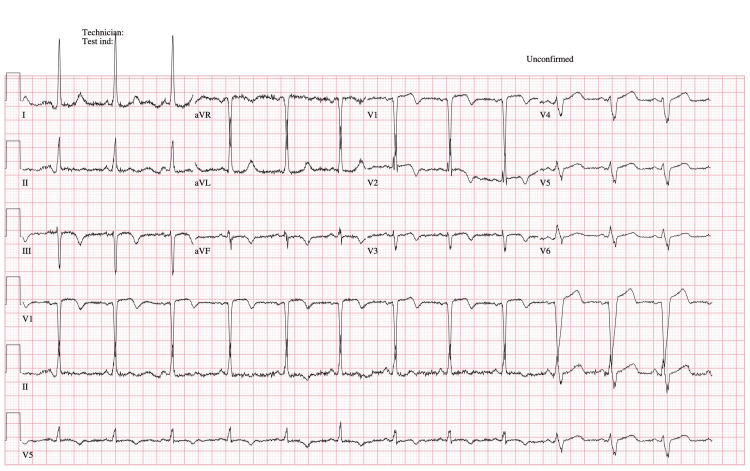
ECG upon ER presentation showing normal sinus ER: emergency room

**Figure 2:**
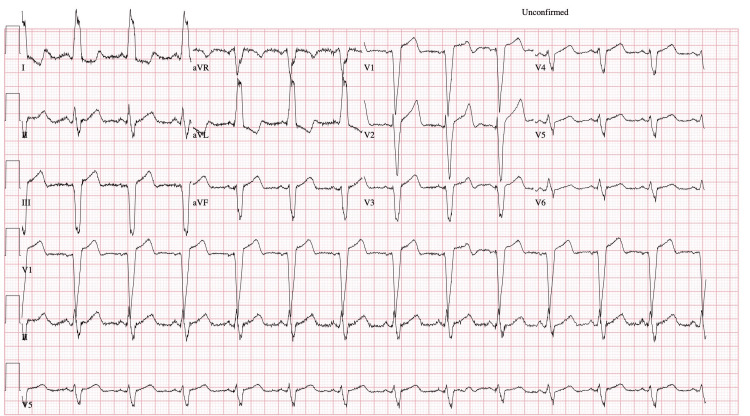
ECG after 26 seconds showing LBBB LBBB: left bundle branch block

The patient's ECG revealed a normal QRS complex alternating with LBBB due to intermittent delay in the left bundle branch. The initial ECG, conducted at 00:36:24, showed normal sinus rhythm with a ventricular rate of 75 bpm and a QRS duration of 72 ms, without LBBB (see Figure 1). The second ECG, performed 26 seconds later at 00:36:50, demonstrated normal sinus rhythm with left axis deviation and LBBB. The ventricular rate was 78 bpm, the QRS duration was 158 ms, and the QTc interval measured 533 ms (see Figure 2).

The patient was referred to the cardiology team with suspected acute coronary syndrome presenting as an ST-segment elevation myocardial infarction (STEMI) equivalent. She received aspirin 300 mg orally and ticagrelor 180 mg tablet for one dose. Subsequently, she underwent urgent coronary angiography, revealing a significant lesion at the most distal segment of the left circumflex artery and a non-significant lesion in the proximal left anterior descending artery (LAD). Cardiac enzyme levels were within normal range, and no ECG was performed. The diagnosis of CAD was confirmed. As a result, the patient was initiated on dual antiplatelet therapy: aspirin 100 mg once daily and ticagrelor 90 mg twice daily for a duration of one year. The patient was also given a clinic follow-up after one month where his condition improved.

## Discussion

Patients with intermittent LBBB experience periods of conduction with LBBB and periods without it [2]. Typically, the diagnosis of LBBB is established through an ECG and is defined by the American College of Cardiology (ACC) and the American Heart Association (AHA) with the following criteria: The rhythm must be of supraventricular origin, QRS duration greater than 120 ms, Lead V1 should exhibit either a QS or a small R wave with a dominant or large S wave, and Lead V6 should have a notched R wave and no Q wave [3].

Moreover, LBBB is found to be prevalent in about 0.06% to 0.1% of the general population, with a frequency of 33% in heart failure patients [3]. Some variants of intermittent LBBB, such as exercise-induced LBBB, occur in nearly 0.38% of all patients undergoing exercise tests.

Transient LBBB is primarily caused by a change in heart rate, such as tachycardia or bradycardia. It can also be triggered by an underlying cardiac pathology, most commonly following myocardial ischemia or blunt cardiac trauma. In certain cases, an ECG examination in the absence of LBBB allows us to identify the underlying condition [2,4].

Transient LBBB can also present with variability and sometimes in unusual situations. There are reported cases in the literature of this phenomenon occurring during anesthesia, cardiac interventions such as catheterization, in cases of acute pulmonary embolism, and even in instances of mad honey poisoning [5].

Moreover, an interesting case was reported involving a middle-aged man who exhibited intermittent LBBB on a baseline ECG. This phenomenon vanished upon coughing and then reappeared with the same maneuver. The patient received IV dipyridamole and IV Tc-99m tetrofosmin for stress single-photon emission computed tomography (SPECT) imaging, along with an IV aminophylline following the protocol for myocardial perfusion imaging. Neither the medications nor the injection resulted in a change from LBBB to a narrow complex rhythm. The patient's management included oral proton pump inhibitors, and he was closely monitored for any symptoms warranting a coronary angiogram. He remained asymptomatic on proton pump inhibitor (PPI), and it was believed that the appearance and disappearance of intermittent LBBB conduction changes were likely due to alterations in vagal tone during coughing, as reflected on the ECG [6]. However, in our case, no specific maneuver was associated with the appearance and disappearance of LBBB.

Another case described transient LBBB as a manifestation of myocardial contusion after blunt chest trauma. This occurred in a medically healthy 38-year-old male involved in a road traffic accident. The only ECG abnormality noticed was LBBB. The patient was stabilized and transferred to the ICU, where he experienced a widening of the QRS complexes without any accompanying symptoms. The ECG revealed a normal sinus rhythm with an LBBB pattern, which spontaneously reverted after approximately 40 minutes. Additionally, he displayed a new LBBB pattern with variable frequencies, not associated with hypotension or other symptoms. The patient underwent immediate investigation, with a troponin level within the normal range. Creatinine phosphokinase levels were exceptionally high due to accompanying skeletal muscle injuries. The patient received a diagnosis of grade two myocardial injury [7]. Similarly, in our case, the occurrence of LBBB was not associated with any new symptoms beyond the presenting ones nor changes in the patient's stability, and the diagnosis was established based on ECG findings.

Furthermore, another case involving a middle-aged woman presenting with cardiac chest pain along with intermittent LBBB on ECG was reported. The patient was taken for an emergent coronary angiogram along with receiving aspirin 325 mg, a loading dose of clopidogrel 600 mg, and a heparin drip. The angiogram did not reveal any coronary artery pathology but did show a moderate to severe LAD vasospasm, which was successfully relieved by intravenous nitroglycerin. Additionally, during chest pain episodes, serial ECGs were consistent with LBBB and demonstrated of a resolved LBBB pattern with deep T wave inversion in Leads V1-V4 during chest-pain-free intervals. Finally, with additional negative cardiac enzyme results and a normal echocardiogram, the patient received a diagnosis of coronary vasospasm and was started on daily diltiazem. This provided her with significant symptomatic relief [1].

Although our patient was 60 years old, hypertensive, and diabetic, which increases the risk of developing LBBB, intermittent LBBB can also occur in healthy individuals. Dennis M. Krikler reported two cases of normal healthy individuals, with no underlying cardiac pathology, who were found to have intermittent LBBB. He concluded that while most patients with LBBB have underlying organic heart disease, there are enough isolated cases in individuals without obvious heart disease to suggest that this abnormality can be benign [8].

Finally, in relation to our case, there is no specific treatment for LBBB; the treatment is mainly directed at treating the underlying disorder. The identification of rate-dependent LBBB is of utmost importance because it needs to be differentiated from new-onset LBBB with ischemic chest pain. In such cases, it is typically treated as a transmural myocardial infarction (MI), where acute revascularization therapy is indicated.

## Conclusions

Little is known about the prognosis of patients with intermittent LBBB. Therefore, patients with LBBB should undergo a thorough medical assessment, as this conduction might be associated with catastrophic cardiac pathologies and could have an adverse effect on ventricular function. Nevertheless, a meticulous analysis of the ECG, with special attention to the onset and offset of LBBB, may provide insights into its functional and benign nature. This poses a challenge for clinical correlation and further investigation to identify the nature and severity of the underlying cause.

There are some challenges encountered during the diagnosis of intermittent LBBB which include the difficulty in capturing the irregular conduction pattern during standard ECGs, leading to intermittent false-negative results. Confounding factors may involve coexisting cardiac conditions or medications affecting conduction, which can complicate the accurate identification and interpretation of intermittent LBBB episodes. These factors underscore the complexity involved in diagnosing and understanding the dynamics of this rare cardiac phenomenon.

## References

[1] Alhaji M (2013). Intermittent left bundle branch block caused by coronary vasospasm. Avicenna J Med.

[2] Abben R, Rosen KM, Denes P (1979). Intermittent left bundle branch block: anatomic substrate as reflected in the electrocardiogram during normal conduction. Circulation.

[3] Scherbak D, Hicks GJ (2023). Left bundle branch block. StatPearls [Internet].

[4] Ameen M (2018). Intermittent left bundle branch block - a challenging case of rare electrocardiogram phenomenon. Eur J Arrhythm Electrophysiol.

[5] Bazoukis G, Tsimos K, Korantzopoulos P (2016). Episodic left bundle branch block-a comprehensive review of the literature. Ann Noninvasive Electrocardiol.

[6] Shahab H, Faheem O, Khandwala K, Khan AH (2018). A curious case of intermittent left bundle branch block associated with cough. Cureus.

[7] Pizzo VR, Beer I, de Cleva R, Zilberstein B (2005). Intermittent left bundle branch block (LBBB) as a clinical manifestation of myocardial contusion after blunt chest trauma. Emerg Med J.

[8] Krikler DM, Lefevre D (1970). Intermittent left bundle-branch block without obvious heart-disease. Lancet.

